# Gastronomics

**Published:** 1876-01

**Authors:** Ben. H. Pratt


### THE BISTOURY.

Vol. XI. ELMIRA, N. Y., JAN., 1876. No. 4.

### Contributions.

### GASTRONOMICS.

BEN. H. PRATT.

We wouldn't for any small consideration, oppose scientific research. Science has done a world of good, and will yet do infinitely more good. In the realm of physics proper, there is no limit to the boon it may confer upon the wisdom and wealth of mankind. But whether it become a science of ultimate weal or woe, depends entirely upon the subjects to which, and objects for which it lends investigation. There are scientists and scientists. There are investigators whose aim is knowledge—knowledge that will ameliorate the race. There are investigators whose aim is glorification—glorification that will resound to an elevation of self. There are searchers after hidden truth and searchers after coveted notoriety. Science, in the hands of the former, is nobly directed, and its results are daily chronicled among our proud achievements; in the hands of the latter it is basely misdirected, and its effects are being already felt in their tendencies to demoralize the body social, and enfeeble the gastronomy of the over credulous.

I tell you, folks are dying of too much gastronomic science. Every doctor that can't make a living in the legitimate pursuit of his profession, either from indisposition, inability, incapacity or laziness, turns to and writes a book on the science of eating. And they are a curious lot, this collection of sophic philosophers, on the subject of preparing food, masticating food, swallowing food, digesting food. A perusal of these would convince the credulous reader that mankind had gone on these six thousand or more years in the blindest ignorance of what they should eat and drink; that men had been born, lived and died, century after century, without the faintest notion of what should go into their stomachs; that it is a matter of pure accident that life hitherto has been sustained from childhood to old age, and the only surprise is that the human race did not become extinct long ago through its ignorance of dietetics. The world has got along somehow—has fed and feasted—waxed fat and strong—built up its bone, blood, nerve, muscle—sustained the exactest relations of its physical and mental conditions—and all in blind ignorance of the great life-sustaining elements of that which has nourished it.

Now we are met on every hand with essays on food and its proper administration. You find works on diet lying upon your library table—treatises on digestion find their way into your book-cases—health by good living and health on oat meal and graham crackers, stare you out of countenance as they lie open and face down on chair and lounge and floor, dropped by the member of your family that always knew there was something wrong in the way the world was gobbling up its miscellaneous nourishment—dropped for a moment to attend to some pressing duty. You begin to be asked much oftener than you used, your opinion of this, that and the other article of food. You are gravely invited to give your views on mush—explain the bile-producing effects of cows' milk—account for the poison in cheese—give a cause for the long life of those who daily imbibe that liquid death known as coffee. The least complaint on your part begets an immediate inquiry into your diet for the last fortnight. It was that roast beef, or that strong coffee, or that piece of pie, or that bit of cheese, or that habit of smoking a pipe after dinner, that caused all the trouble. You don't deserve any sympathy because you won't eat potatoes and salt and change off occasionally with graham mush and oat meal porridge. If you are uncomfortably fat, you are preached at continually because you will persist in using the *carbonates* as food; if wretchedly lean, you are being scolded for allowing yourself to indulge in the *nitrates;* if you are as stupid as a fool, you hear folks wonder why that thick headed old ass don't eat things that contain the *phosphates*; if you are neither too fat nor too lean, and have a reasonably clear head, folks wonder how it is you manage it, eating such food as you do.

These diet bibliographers, like the patent medicine men, understand human nature. They know the weaknesses of humankind, and are taking advantage of them. They have observed that nine people out of ten are out of sorts from some cause or or another. Now, if they can induce people to believe that their ailments are the outgrowth of a wrong system of eating and drinking—which seems to be no difficult task—and can trump up some great radical change in the daily food-taking, bringing to bear a long line of sophistry in support of their peculiar views, they can readily catch the ear of well nigh everybody; for there is no subject upon which people are so lamentably ignorant as upon their own functions of life; that is, upon the effect of what is introduced within their bodies in the way of nourishment, and the *modus operandi* of the various organs and fluids of the human system upon that which is so introduced, while it is being converted into the constituent elements which go to make up bone and muscle and blood and nerve, and supply the daily wastes thereof. The mystery of life, whether it be animal or vegetable, is so deep an one that none have ever fathomed it. Philosophy has been investigating the active principle of life ever since the world began, and big minds and little ones have been puzzling over it, instituting theory after theory, and building thereon system after system, in the vain hope of arriving at some fixed and settled doctrine which shall enable men to fashion their forms and direct their functions with an absoluteness beyond peradventure. This very mystery is the great attraction; and it is because nobody knows anything about it, and is likely never to know anything about it, that men can impose upon their kind by starting up every now and then a good theory, which they feel safe in sustaining simply because there are no means at hand of refuting it. And the civilized world, ever greedy to accept something in lieu of nothing—to adopt somebody's opinion because they have no settled one of their own—to try everything in the hope of finding something that will meet their case—seize upon every new fangled theory that is hurled at them through a printed book, and try to live by rule. And living by rule is a good deal like singing by note without any *ad libs* or *crescendos* or *diminuendos* or *fortes* or *pianos* or *holds*—it's sure death to the music and excruciating torture to the hearer. But people will be so foolish as to believe that such and such articles of food only are admissible into the system—that such and such quantities per diem can be measured out—that just so much time must be employed in performing the offices of mastication, deglutition, assimilation and digestion—that, in a word, eating must be reduced to an exact science and conducted with a pair of scales on one side of your plate and a gill measure on the other, with a thermometer and a chronometer between the two, and a foot rule and a magnifying glass handy, as necessary to test quantities and qualities. It is presupposing that all men and women are constituted precisely alike, and that all stomachs and livers and bowels are exact counterparts of each other—that the blacksmith and the preacher and the sewing woman require precisely the same quantity and quality of food—that all the infinite variety of grains and vegetables, fruits, nuts, beasts, birds and fishes were created simply to please the eye and tempt mankind to eat that which is hurtful—that appetite and taste and varied temperament and climate and occupation and condition, don't matter—that here is so much of just such which you must eat of and live, and he that eateth not by the square and rule and scales and measure, shall surely suffer the torments of the belly, so long as he shall continue to drag along his miserable existence.

We don't see it. Try it, if you like. Go hungry and keep your stomach at the craving point all the time. Sit down to the table ravenous for some nourishing, palatable, strengthening food—deny yourself continually—get up from the table as hungry as you sat down—and you'll be chuck full of the consciousness that you've done your whole duty to Dio Lewis, or some other hnmbug, if you have to die for it.

Keep your mind on your stomach. Study up your stomach—its anatomy and physiology—its functions and its attributes. Dwell upon it continually. Imagine how it looks when it's full and when it's empty—how it swells and how it collapses—how, may be, there's something growing in it, tape worm, or other gentle creature, that is making his habitation inside of it and having a good time there all alone in the dark. Keep your mind on it and what goes into it—and if you don't get sick and have a long spell of something, it will be your own fault. Half the people who are ailing are in your fix precisely. They are trying to square their diet by somebody's silly notions and are getting the benefit of it.

We don't pretend to refute Dio Lewis' plan of living; we only say, try it—go ahead; one might as well die o' Lewis as die of anything else. You can't coax or drive people from these isms and ologies and ics. Give them all the rope they want, and our word for it, five years hence there'll be such an army of stomach-robbed men and women in this country that Dio Lewis, and all his ilk, will call upon the rocks and the hills to fall upon them and hide them forever from the ghostly visitations of those whose stomachs they have ruined.

